# Accelerometer measurement error in a randomized physical activity intervention trial in breast cancer survivors was nondifferential but attenuated the intervention effect

**DOI:** 10.1186/s12966-025-01760-5

**Published:** 2025-05-26

**Authors:** Laura Q. Rogers, Douglas Midthune, Kevin Dodd, Heather Bowles, Edward McAuley, Kerry S. Courneya, Brian Barrett, Spiro Razis, Gary R. Hunter, Stephen J. Carter, Raymond J. Carroll, Victor Kipnis

**Affiliations:** 1 Division of General Internal Medicine and Population Science, Department of Medicine, Heersink School of Medicine, University of Alabama at Birmingham, 1720 2ndAve S, MT 614, Birmingham, AL 35294-4410, Birmingham, AL USA; 2 O’Neal Comprehensive Cancer Center at UAB, Birmingham, AL USA; 3 Biometry Research Group, Division of Cancer Prevention, National Cancer Institute, Bethesda, MD USA; 4 Department of Health and Kinesiology, University of Illinois at Urbana-Champaign, Urbana, IL USA; 5 The Cancer Center at Illinois, Urbana, IL USA; 6 Faculty of Kinesiology, Sport, and Recreation, University of Alberta, Edmonton, AB Canada; 7 Information Management Services, Inc., Calverton, MD USA; 8 Department of Nutrition Sciences, School of Health Professions, University of Alabama at Birmingham, Birmingham, AL USA; 9 Department of Kinesiology, School of Public Health – Bloomington, Indiana University, Bloomington, IN USA; 10 Department of Statistics, Texas A&M University, College Station, TX USA

**Keywords:** Oncology, Exercise, Differential error, Indirect calorimetry, Intervention trials, Measurement error

## Abstract

**Background:**

Physical activity reduces morbidity and mortality risk in cancer survivors, but a meaningful proportion of this vulnerable population are physically inactive. Targeted interventions can help cancer survivors adopt a more active lifestyle, but the efficacy of these interventions must be rigorously evaluated in randomized controlled intervention trials. A major barrier to such trials involves the difficulty in obtaining unbiased estimates of physical activity in free-living conditions.

**Methods:**

We conducted a randomized controlled trial of a 3-month intervention designed to increase physical activity vs. usual care in breast cancer survivors (n = 316). The primary outcome was change in physical activity as estimated by hip-worn accelerometer (MTI/Actigraph, models GT1M and GT3X). The trial included a sub-study (n = 106) wherein unbiased measures of total energy expenditure (doubly labeled water), and resting energy expenditure (indirect calorimetry) were collected. A linear mixed measurement error model characterized the structure of measurement error in accelerometry-estimated physical activity energy expenditure (PAEE), and corrected for bias in the estimated intervention effect due to measurement error.

**Results:**

Bias in the accelerometer estimates was related to true PAEE (*p* < 0.001) and baseline body mass index (*p* < 0.001) but was not related to age (*p* = 0.13). After correcting for measurement error, the estimated intervention effect at 3 months (change from baseline in PAEE in the intervention arm minus change in the control arm) was 77 kcal/day (95% confidence interval (CI) = 31–125), compared to 48 kcal/day (95% CI = 22–75) when measurement error was ignored. These results indicate a 20% (21%) increase in PAEE kcal x d^−1^ (kcal x kg^−1^ × d^−1^) at month 3 relative to baseline for the corrected model vs. 14% (15%) for the uncorrected model. There was no evidence that measurement error in accelerometry-estimated PAEE was differential (differed by treatment arm) in the trial (p = 0.86).

**Conclusions:**

Measurement error in accelerometer-estimated PAEE can attenuate the effect size related to intervention effects in randomized controlled trials of physical activity interventions. Sub-studies that collect unbiased measures of PAEE can be used to correct for this short-coming.

**Trial registration:**

ClinicalTrials.gov; NCT00929617; registered 06/26/2009; https://clinicaltrials.gov/study/NCT00929617

**Supplementary Information:**

The online version contains supplementary material available at 10.1186/s12966-025-01760-5.

## Background

Physical activity can reduce risk of mortality, cancer recurrence, and co-existent medical comorbidities in cancer survivors [1–3]. Because most cancer survivors do not experience these benefits due to physical inactivity [4, 5], interventions that successfully assist cancer survivors in adopting and maintaining a physically active lifestyle are critical. Rigorous evaluation of the effectiveness of these interventions, however, is hampered by the difficulty in accurately assessing changes in physical activity. Randomized controlled intervention trials have used both self-report and accelerometer to measure physical activity [6]. Self-report physical activity is used more often due to logistical simplicity and lower cost while accelerometer-measured (i.e., estimated) physical activity is considered objective and thus a more scientifically rigorous approach [6]. Yet, both self-report and accelerometer-estimated physical activity have substantial measurement error that can interfere with interpretation of intervention effects on physical activity.

The simplest and least problematic type of measurement error is “classical” error, which includes only random within-subject variation. Measurements with classical error are unbiased estimates of the true value but have extra variability, which leads to a loss of precision in estimated intervention effects and loss of power to detect significant effects but does not otherwise affect intervention studies [7]. Nonclassical error includes systematic and person-specific (subject-level) error in addition to within-subject error, which can lead not only to loss of precision and power but also to bias in the estimated intervention effects [8]. Validation studies using doubly labeled water (DLW) to measure total energy expenditure (TEE) have shown that accelerometry-estimated energy expenditure has both systematic and person-specific error [9, 10].

Another type of measurement error is “differential” error, which occurs when participants in the intervention arm report their physical activity differently than those in the control arm [8]. Self-report measures are particularly prone to this differential error, possibly due to social desirability or other psychosocial factors, which can lead to spurious or inflated intervention effects [11, 12]. Although accelerometer-estimated physical activity is unlikely to be affected by psychosocial factors, it may be affected by other factors that could potentially lead to differential error. We have reported that accelerometer measurement of physical activity may be influenced by improvements in fitness (i.e., greater vertical accelerations associated with lower energetic cost of walking), leading to possible over-estimation of energy expenditure as fitness improves [13]. Others have reported that accelerometry measurements may under-estimate energy expenditure in overweight/obese individuals [14]. These findings suggest that substantial changes in fitness or weight could lead to under- or over-estimation of changes in physical activity over time unless these variables are included as covariates in a measurement error model. This, in turn, could lead to differential error if the changes are greater in one arm than another. Because measurement error reduces precision and increases the risk of inaccurate inferences, a better understanding of the role of accelerometer-based measurement error is warranted.

Our goal was to evaluate the effect of measurement error in a randomized physical activity intervention trial when the outcome (change in physical activity) is measured by accelerometry. To do so, we conducted a biomarker sub-study as part of the Better Exercise Adherence after Treatment for Cancer (BEAT Cancer) study, a multicenter randomized controlled trial testing a 3-month physical activity behavior change intervention for breast cancer survivors [15, 16]. In addition to the accelerometer measurements collected on all participants, sub-study participants also provided unbiased measurements of TEE, measured by DLW (TEE_*DLW*_), and resting energy expenditure (REE), measured by indirect calorimetry (REE_*IC*_). These measurements were combined to create an approximately unbiased measure of physical activity energy expenditure (PAEE).

The sample of breast cancer survivors enrolled in BEAT Cancer was ideally suited for a longitudinal calibration sub-study because the notable declines in physical activity, cardiorespiratory fitness, and physical functioning that often occur with cancer and its treatment are more likely to improve with the intervention when compared with the general population or conditioned athletes who are already near the peak of their cardiopulmonary capacity [17–20]. Moreover, the BEAT Cancer intervention has shown efficacy related to increasing cardiorespiratory fitness (potentially altering accelerometer measurement error over time due to changes in movement profiles and energy expenditure) and longer-term intervention effects demonstrate a discrepancy between the self-report and accelerometer physical activity effects (suggesting greater precision is needed for drawing accurate conclusions about intervention effects).

Previous studies have used DLW and prediction equations for REE to evaluate the structure of measurement error in accelerometer-derived estimates of usual or long-term average TEE and PAEE in an observational setting [9, 10]. To our knowledge, our study is the first to integrate longitudinal measurements of DLW and REE in an intervention trial to evaluate measurement error in short-term PAEE and measured changes over the course of the intervention. In addition, our study used indirect calorimetry to measure REE, while the previous studies used prediction equations that have Berkson error and are not unbiased [7]. Our study aims were as follows: 1) to evaluate and characterize the structure of measurement error in accelerometer-derived estimates of short-term PAEE in an intervention trial, including the possibility that the error is differential; and 2) to evaluate and adjust for bias in the estimated intervention effect due to outcome measurement error in a randomized longitudinal physical activity intervention trial.

## Methods

### Study design

#### The BEAT cancer study

The BEAT Cancer study is a randomized trial of a 3-month health behavior change intervention (BEAT Cancer) designed to increase physical activity in breast cancer survivors. The design of the BEAT Cancer study (including a detailed intervention description) has been previously published [15, 16, 21]. Briefly, 222 post-primary treatment breast cancer survivors with history of ductal carcinoma in situ or Stage 1 through 3 breast cancer were recruited between January 2010 and September 2013 and randomized to either the BEAT Cancer intervention or usual care (i.e., written materials). Participants’ physical activity was measured four times over the course of a year: at baseline (prior to intervention), 3 months (end of intervention), 6 months and 12 months. Physical activity was assessed by the MTI/Actigraph accelerometer (models GT1M and GT3X) that participants were asked to wear for 7 days. The primary outcome of the study was weekly minutes of moderate-to-vigorous physical activity at 6 months as measured by the accelerometer, where moderate-to-vigorous activity is defined as ≥ 1952 counts/minute.

#### The doubly labeled water calibration sub-study

After the BEAT Cancer study met its recruitment goals, we initiated a follow-on doubly labeled water (DLW) calibration sub-study (also known as COMPARE—Comparing doubly labeled water to accelerometer to assess physical activity measurement error during and after a physical activity behavior change intervention). The DLW calibration sub-study recruited 118 participants from September 2014 to May 2016 and followed the same protocol as the BEAT Cancer study but included two additional measures of energy expenditure at each of the four assessment time points: DLW to measure total energy expenditure (TEE) (kcal/day) over a 10-day period, and resting energy expenditure (REE) (kcal/day) measured by indirect calorimetry. The DLW calibration sub-study also extended the accelerometer wear time to 10 days (i.e., for consistency with the 10-day DLW assessment). DLW and indirect calorimetry have been shown to provide unbiased measures of TEE and REE at the individual level [22–24].

#### Samples analyzed

**Fig. 1:**
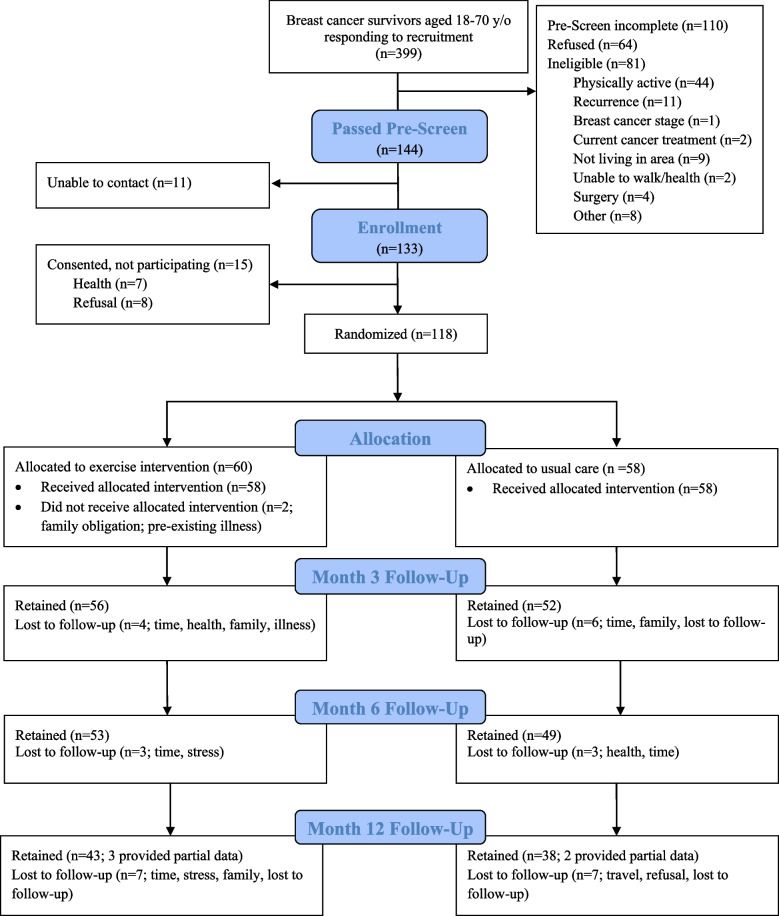
Participants’ flow through the doubly labeled water (DLW) calibration sub-study. More than one reason for ineligibility was possible

We analyze data from 290 study participants, including 208 participants from the original BEAT study and 82 who participated in the DLW calibration sub-study and had at least one follow-up measurement of DLW and REE. When combining BEAT Cancer and DLW calibration sub-study data sets, only BEAT Cancer data was used for the 21 participants who participated in both studies. A CONSORT (consolidated standards of reporting trials) diagram has previously been published for the BEAT Cancer study [15] with a CONSORT diagram provided here for the DLW calibration sub-study (Fig. 1; also see Additional File 1 for CONSORT checklist). Nine participants enrolled in the final DLW calibration sub-study participant “wave” or cohort were not followed up at month 12 because of the funding period end date. Table 1 provides the number of participants available for the analyses reported here based on removal of duplicates and available data.

**Table 1:** Definition of the 290 participants used in accelerometer calibration analyses

	**Study source**		
	**BEAT Cancer**	**Doubly labeled water calibration sub-study**	**Total**
1. All participants (includes duplicates)	222	118	340
2. Duplicate participants removed	222	98	320
3. Demographics and valid accelerometer^a^ available at baseline	214	91	305
4. Valid accelerometer available at other time points	208	82	290

^a^Valid accelerometer defined as 4 + valid days and daily calories variable available; valid day defined as at least 8 h of accelerometer data per day

#### Measures

We compare PAEE in the intervention and usual care arms, using TEE measured by DLW (TEE_*DLW*_) and REE measured by indirect calorimetry (REE_*IC*_) as reference biomarkers to correct for measurement error in accelerometry-estimated PAEE. Free-living TEE was estimated using 10-day DLW measurement as previously described [25]. In brief, fasting participants provided a baseline urine sample, ingested an oral dose of ^2^H_2_O and H_2_^18^O based on body weight, and provided 3- and 4-h post-dose urine samples (for isotopic equilibrium). Participants then provided two morning urine samples 10 days later. TEE was calculated as 3.9 × (rate of carbon dioxide production/respiratory quotient) + 1.1 [25, 26]. The REE component of PAEE was assessed with a ventilated canopy and indirect calorimetry using Vmax Encore (Loma Linda, CA, USA) for 30 min (10 min allowed for equilibrium followed by 20 min for the steady-state measurement) [25]. To optimize reliability of the REE measure, participants completed the test fasting and during the same time of day (i.e. morning). Further, we used a ventilated-hood system which has a lower within-individual coefficient of variation compared to a mouthpiece/nose-clip system [27] and the analyzer units were calibrated with primary standard calibration gas supplied by the manufacturer before testing each patient. Quarterly precision ethyl alcohol combustion tests were run according to the manufacturer’s instructions, and all test results were within the manufacturer specification for the Vmax Encore system. The respiratory quotient for precision alcohol combustion is 0.67, and all combustion tests run on the REE carts were within ± 1.5% of a respiratory quotient of 0.67. With regard to diet-induced thermogenesis, budgetary constraints prevented calculating precise estimates from diet measures thus diet-induced thermogenesis was estimated as successfully done in our prior studies [28].

Accelerometer-assessed physical activity was performed using a hip-worn Actigraph. The DLW calibration sub-study used a GT3X exclusively while BEAT Cancer transitioned from a GT1M to the GT3X once available; both studies ensured that the same accelerometer was used for all assessments for a participant because of our focus on within person change. The accelerometer wear protocol has been previously described for BEAT Cancer [16]; the DLW calibration sub-study used the same protocol with the exception of extending the wear time from 7 to 10 days. PAEE was based on daily kilocalories generated by the Actigraph software.

Participants were instructed to record time they went to bed to sleep and time awake on an accelerometer log sheet which was double checked by a study staff member, reviewed for consistency with the graphical presentation of accelerometer counts provided with the ActiLife® software, and entered into the software before accelerometer scoring. Sleep time was excluded from the PAEE analyses and accounted for in the REE because of the minimal amount of activity-related EE during sleep. Although this may slightly underestimate daily PAEE, it reflects the typical calculation of PAEE in physical activity intervention trials (increasing relevance of the analyses) and our focus is on within person changes over time rather than error in a cross-sectional sample.

Also as previously described [16], participant characteristics were assessed via self-administered survey (age, race, ethnicity, cancer stage, cancer treatment history, and months since breast cancer diagnosis). Body mass index (BMI = weight [kg]/height [m^2^]) was assessed by trained research staff using stadiometer and scale with participants in-person and wearing light clothing without shoes. Cardiorespiratory fitness was estimated using a submaximal treadmill test consistent with the modified Naughton protocol as previously described. Peak oxygen uptake (ml/kg/min) was estimated based on the workload at which the participant reached 85% of age-predicted maximum heart rate using regression equations provided in the American College of Sports Medicine’s Guidelines for Exercise Testing and Prescription [29].

#### Physical activity metrics

In our analysis, we used daily PAEE (kcal × d^–1^) and PAEE scaled for body mass, i.e., divided by kilograms of body weight (kcal × kg^–1^ × d^–1^), to evaluate possible intervention effects. These metrics are commonly used in physical activity studies and have the advantage that they can be estimated using biomarkers. The biomarker estimate of PAEE (kcal × d^–1^) is PAEE_DLW_ = 0.9 × TEE_DLW_ – REE_IC_. In this equation, TEE_DLW_ is multiplied by 0.9 to adjust for the thermic effect of food, which accounts for about 10% of TEE [7]. REE was measured while awake and scaled up to represent a 24-h period, resulting in a small negative bias in estimated PAEE due to the fact that energy expenditure during sleep is typically about 5% lower than REE while awake. Despite these approximations, we expect PAEE_DLW_ to provide an approximately unbiased estimate of PAEE.

Accelerometer counts were recorded in 1-min epochs, and the ActiLife® software was used to automatically convert accelerometer counts per minute (CPM) and body mass (BM) (kg) to estimated PAEE kcal per minute (KPM) based on the “Freedson Combination (98)” algorithm [30], defined as follows:

$$CPM\hspace{0.17em}\le \hspace{0.17em}1951: KPM=CPM\times BM\times 1.91\times {10}^{-5}$$

$$CPM\hspace{0.17em}>\hspace{0.17em}1951: KPM=Scale\times \{CPM\times 9.4\times {10}^{-4}+BM\times 0.1346-7.37418\}$$

### Statistical analysis

#### Intervention model

Let *X*_*ij*_ be the unobserved true PAEE (kcal/day) for subject *i* at time point *j*, *i* = 1 to *N*, *j* = 1 to 4. Time points 1 to 4 correspond to months 0 (baseline), 3 (end of intervention), 6 and 12, respectively. We assume a linear mixed model of the effect of the intervention on PAEE over time,

1 $${X}_{ij}={\beta }_{0}+{\beta }_{A}{A}_{i}+{{\varvec{\upbeta}}}_{T}^{\prime}{\mathbf{T}}_{j}+{{\varvec{\upbeta}}}_{TA}^{\prime}{\mathbf{T}}_{j}{A}_{i}+{{\varvec{\upbeta}}}_{Z}^{\prime}{\mathbf{Z}}_{i}+{u}_{Xi}+{\varepsilon }_{Xij}$$

where* A*_*i*_ is study arm (0 = usual care, 1 = BEAT Cancer intervention), **T**_j_ is a 3 × 1 vector of indicator (dummy) variables for time points 2–4, ${\mathbf{Z}}_{i}$ is a vector of subject-level covariates, ${u}_{Xi}$ is a subject-level random intercept, and ${\varepsilon }_{Xij}$ is random within-subject deviation. Covariate vector ${\mathbf{Z}}_{i}$ can be used to adjust for any imbalances between the arms at baseline after randomization. In our analysis, ${\mathbf{Z}}_{i}$ includes age and the log of BMI at baseline. We assume that ${u}_{Xi}$ and ${\varepsilon }_{Xij}$ are mutually independent and normally distributed with means equal to zero and variances ${\sigma }_{{u}_{X}}^{2}$ and ${\sigma }_{{\varepsilon }_{X}}^{2}$, respectively. If ${X}_{ij}$ were observed, model (1) could be fit using standard mixed model software.

The vector of regression coefficients ${{\varvec{\upbeta}}}_{T}$ = ${(\beta }_{T2,}{\beta }_{T3,}{\beta }_{T4}){\prime}$ in model (1) represent the change from baseline to time points 2, 3 and 4 (months 3, 6, and 12) in the usual care arm. The inclusion of the interaction term ${\mathbf{T}}_{j}{A}_{i}$ in model (1) allows mean PAEE to differ between the two study arms at time points 2–4 (months 3, 6, and 12), with vector ${{\varvec{\upbeta}}}_{TA}=({\beta }_{TA2},{\beta }_{TA3},{\beta }_{TA4}){\prime}$ representing the intervention effect at each time point. Additionally, the inclusion of study arm (*A*_*i*_) in the model allows mean PAEE to differ at baseline. Since this is a randomized trial, any difference in means prior to intervention should be due to random variation, so we also consider a reduced model that assumes ${\beta }_{A}=0$, which constrains the means in the two arms to be equal at baseline. The reduced model provides more efficient estimates of intervention effects when the two arms have equal means at baseline, or any difference is due to random variation [31]. Following Liu et al. [32], we refer to model (1) as the ‘longitudinal data analysis’ (LDA) model, and the reduced model that assumes ${\beta }_{A}=0$ as the ‘constrained’ LDA (cLDA) model.

The LDA and cLDA models are closely related to the ‘change score’ and ‘ANCOVA’ (Analysis of covariance) methods commonly used in intervention trials [33]. When there are no missing data, the change score and LDA estimates of the intervention effects are identical, as are the ANCOVA and cLDA estimates [31, 32]. When data are missing, however, the LDA and cLDA models are able to use all the available data and provide more efficient estimation of the effects than the change score and ANCOVA methods. In addition, when data are missing at random (MAR), the change score and ANCOVA methods can produce biased estimates of the intervention effects, while the LDA and cLDA models still provide consistent (asymptotically unbiased) estimates [32]. See the online supplemental materials for details of the change score and ANCOVA methods.

#### Measurement error model

When the outcome of an intervention trial has systematic measurement error, special methods are required to avoid biased results. Let ${W}_{ij}$ be accelerometry-estimated PAEE, and let ${M}_{ij}$ = 0.9 × TEE_*DLW*_ – REE_*IC*_ be the reference biomarker for PAEE. We assume that ${M}_{ij}$ has classical measurement error but that ${W}_{ij}$ has systematic error that depends on ${X}_{ij}$. The measurement error models for ${M}_{ij}$ and ${W}_{ij}$ are

2 $${M}_{ij}={X}_{ij}+{e}_{Mij}$$

3 $${W}_{ij}={\alpha }_{0}+{{\varvec{\upalpha}}}_{Z}^{\prime}{\mathbf{Z}}_{i}+{\alpha }_{X}{X}_{ij}+{u}_{Wi}+{e}_{Wij}$$

where ${u}_{Wi}$ is a person-specific (subject-level) bias and ${e}_{Wij}$ and ${e}_{Mij}$ are within-subject random errors. We assume that ${u}_{Wi}$, ${e}_{Wij}$ and ${e}_{Mij}$ are mutually independent and normally distributed with means equal to zero. Variances $var({u}_{Wi})={\sigma }_{{u}_{W}}^{2}$ and $var\left({e}_{Mij}\right)={\sigma }_{{e}_{M}}^{2}$ are assumed constant over *i* and *j*, while $var\left({e}_{Wij}\right)={\sigma }_{{e}_{W}}^{2}/{n}_{ij}$ depends on the number of valid days (*n*_*ij*_ = 4–11) the accelerometer was worn during that time period (valid day defined as at least 8 h of accelerometer data per day). In model (3), *W*_*ij*_ has systematic error unless α_*X*_ = 1. A critical assumption of the model is that measurement error in the biomarker M_ij_ is “classical”, that is, includes only random within-subject variation. The DLW method and indirect calorimetry have been studied extensively and been shown to provide unbiased estimates of TEE and REE [34, 35]. Approximating the thermogenic effect of diet as 0.1 × TEE will introduce a small subject-level bias but should not substantially affect results.

We use the change score method to illustrate the effect of measurement error. Let ${D}_{Xij} ={X}_{ij}-{X}_{i1}$, ${D}_{Wij} ={W}_{ij}-{W}_{i1}$ and ${D}_{Mij} ={M}_{ij}-{M}_{i1}$ be the change scores for their respective measures. Under models (2) and (3), *D*_*Mij*_ = *D*_*Xij*_ + (*e*_*Mij*_ − *e*_*Mi*1_) is an unbiased estimator of ${D}_{Xij}$, while *D*_*Wij*_ = α_*X*_*D*_*Xij*_ + (*e*_*Wij*_ − *e*_*Wi*1_) is biased unless α_*X*_ = 1. As a result, intervention effects estimated using *M*_*ij*_ will be unbiased, while effects estimated using *W*_*ij*_ will have a multiplicative bias equal to α_*X*_.

Measurement error also causes loss of power to detect intervention effects. If a trial in which true change score ${D}_{Xij}$ is observed required N subjects (N/2 in each arm) to have sufficient power to reject the null hypothesis of no intervention effect, then a similar trial in which ${D}_{Wij}$ is observed would require *N*^*W*^ = *N*/${\rho }_{{D}_{X}{D}_{W}}^{2}$ subjects to reject the same hypothesis with the same power, where ${\rho }_{{D}_{X}{D}_{W}}$ is the partial correlation of ${D}_{Xij}$ and ${D}_{Wij}$ controlling for study arm *A*_*i*_. See the online supplemental material for details. If ${\rho }_{{D}_{X}{D}_{W}}$ is small, the loss of power can be profound. For example, if ${\rho }_{{D}_{X}{D}_{W}}$ = 0.2, the sample size would need to be increased by a factor of twenty-five (1/${\rho }_{{D}_{X}{D}_{W}}^{2}$) to make up for the loss of power due to measurement error.

In our analysis, we estimated the intervention effects (β_*TA*_) by fitting a structural model of the joint distribution of ${W}_{ij}$, ${M}_{ij}$ and ${X}_{ij}$, defined by the combined model (1)-(3). This approach provides consistent estimates as long as the model is correctly specified. Keogh et al. [8] considered a similar model and performed simulations showing that classical measurement error in the biomarker affects the precision of the estimated intervention effects but does not lead to bias or invalidate statistical inference. They also showed that an estimator that combines the information in $W_{ij}$  and $M_{ij}$  using the structural model (1)-(3) is more efficient than one based on $M_{ij}$  alone, particularly when $W_{ij}$  has non-differential error. By combining the information in ${W}_{ij}$ and ${M}_{ij}$, it also minimizes the loss of power due to measurement error. In our analysis, we fitted the model using the full information maximum likelihood (FIML) method implemented in the SAS Calis procedure (SAS Institute Inc, version 9.4).

Prior to fitting the model, ${W}_{ij}$ and ${M}_{ij}$ were transformed to better approximate normality, using a log transformation for ${W}_{ij}$ and a square root transformation for ${M}_{ij}$. Unobserved ${X}_{ij}$ is assumed to be on the same scale as ${M}_{ij}$ (square root), since ${M}_{ij}$ has classical error. Also prior to modeling, observed variables ${W}_{ij}$, ${M}_{ij}$, and ${\mathbf{Z}}_{i}$ were standardized to have mean = 0 and variance = 1 at baseline (*j* = 1). Standardization helps stabilize the model fitting process and aids interpretation of the regression parameters when variables are transformed.

#### Differential measurement error

Model (3) in the previous section assumes that measurement error in ${W}_{ij}$ is nondifferential, i.e., that the bias in ${W}_{ij}$ does not depend on the study arm to which a subject is randomized. If this assumption is violated, fitting the structural model (1)-(3) described in the previous section can lead to biased estimation of intervention effects [8]. In this section, we consider models that account for differential error.

Differential error is typically due to a time-varying covariate (${F}_{ij}$) that is affected by the intervention and is also correlated with random error ${e}_{Wij}$ in model (3). This relationship can be modeled as follows,

4 $${F}_{ij}={\gamma }_{0}+{\gamma }_{A}{A}_{i}+{{\varvec{\upgamma}}}_{T}^{\prime}{\mathbf{T}}_{j}+{{\varvec{\upgamma}}}_{TA}^{\prime}{\mathbf{T}}_{j}{A}_{i}+{{\varvec{\upgamma}}}_{Z}^{\prime}{\mathbf{Z}}_{i}+{u}_{Fi}+{\varepsilon }_{Fij}$$

5 $${W}_{ij}={\alpha }_{0}+{{\varvec{\upalpha}}}_{Z}^{\prime}{\mathbf{Z}}_{i}+{\alpha }_{X}{X}_{ij}+{\alpha }_{F1}{F}_{i1}+{\alpha }_{F}({F}_{ij}-{F}_{i1})+{u}_{Wi}+{e}_{Wij}$$

where ${u}_{Fi}$ is correlated with ${u}_{Xi}$ and ${\varepsilon }_{Fij}$ is correlated with ${\varepsilon }_{Xij}$. Under this model, error is differential if ${{\varvec{\upgamma}}}_{TA}\ne 0$ and ${\alpha }_{F}\ne 0$. The cLDA version of model (4) assumes ${\gamma }_{A}=0.$

If ${F}_{ij}$ is known and observed in the trial, fitting a structural model of the joint distribution of ${W}_{ij}$, ${M}_{ij}$, ${X}_{ij}$ and ${F}_{ij}$, defined by models (1)-(2) and (4)-(5), will provide consistent estimates of the intervention effects as long as the model is correctly specified. In our analysis, we use this approach to look for evidence of differential error due to BMI or cardiorespiratory fitness.

If differential error is suspected but the source is unknown or unobserved, a more general approach is to fit model (3) separately in each study arm. This more general approach, though, can lead to substantial loss of precision and power [8].

## Results

Table 2 presents participant characteristics by intervention arm for the variables and 290 participants included in the current analyses. Details regarding the full sample of 320 participants can be found in a prior publication [36]. The estimated correlation between repeated measurements of REE was 0.89.

**Table 2:** Baseline characteristics overall and by study group for the participants included in the calibration analyses

Variable	Overall (*n* = 290)	Intervention (*n* = 148)	Usual care (*n* = 142)
Age in years (Median (IQR^a^))	56.0 (10.0)	56.0 (11.0)	55.0 (10.0)
Body mass index (kg/m^2^) (Median (IQR^a^))	30.1 (9.1)	30.7 (10.3)	29.9 (7.6)
Cardiorespiratory fitness (ml/kg/min) (Median (IQR^a^))	19.0 (3.4)	20.7 (3.4)	19.0 (7.0)
Cancer stage (no. (%))			
DCIS^b^	36 (12%)	18 (12%)	18 (13%)
1	115 (40%)	61 (41%)	54 (38%)
2	111 (38%)	54 (36%)	57 (40%)
3	28 (10%)	15 (10%)	13 (9%)
Current hormonal therapy (no. (%))			
None	142 (49%)	76 (51%)	66 (46%)
Aromatase inhibitor	79 (27%)	34 (23%)	45 (32%)
Estrogen receptor modulator	69 (24%)	38 (26%)	31 (22%)
Baseline PAEE kcal × d^–1^ (Mean (SD^c^))			
Accelerometer	429 (194)	432 (211)	426 (176)
Biomarker (sub-study)	502 (249)	523 (261)	479 (236)
Baseline PAEE kcal × kg^–1^ × d^–1^ (Mean (SD^c^))			
Accelerometer	5.14 (1.73)	5.18 (1.88)	5.11 (1.58)
Biomarker (sub-study)	6.23 (3.00)	6.44 (3.15)	5.97 (2.82)

^a^IQR = Interquartile range (difference between the 75 th and 25 th percentiles of the data)

^b^DCIS = Ductal carcinoma in situ

^c^SD = Standard deviation

Table 3 presents estimated parameters for the measurement error models (2)-(3) in the BEAT/DLW calibration sub-study, assuming nondifferential measurement error in ${W}_{ij}$. Results for absolute PAEE (kcal × d^−1^) are on the left side of the table, while results for PAEE scaled for body mass (kcal × kg^−1^ × d^−1^) are on the right. For absolute PAEE, the estimated regression coefficients ($\alpha$) indicate that measurement error in accelerometry-estimated PAEE does not depend on age (*p* = 0.167) but does depend on BMI and true PAEE ${X}_{ij}$ (*p* < 0.001). The estimated coefficient ${\alpha }_{X}=0.568$ for absolute PAEE represents bias in the estimated intervention effect due to measurement error, meaning that a unit change (one standard deviation) in ${X}_{ij}$ results in only about a half-unit change in *W*_*ij*_. Estimated correlation ${\rho }_{{D}_{X}{D}_{W}}=0.568$ represents the loss of power to detect a nonzero intervention effect, meaning that a trial using ${W}_{ij}$ to measure PAEE would need about $1/{\rho }_{{D}_{X}{D}_{W}}^{2}\approx 3$ times as many subjects as a trial using ${X}_{ij}$. The results for body-mass-scaled PAEE are similar, although the estimated coefficient ${\alpha }_{X}=0.696$ indicates less bias due to measurement error in the estimated intervention effect.

**Table 3:** Estimated parameters for the measurement error models^a^ for physical activity energy expenditure (PAEE) in the BEAT/doubly labeled water calibration sub-study

Parameter	PAEE kcal × d^–1^		PAEE kcal × kg^–1^ × d^–1^	
	Estimate (s.e.)	*p*-value	Estimate (s.e.)	*p*-value
${\alpha }_{0}$	0.151 (0.065)	0.020	0.205 (0.082)	0.013
${\alpha }_{Z1(Age)}$	−0.093 (0.068)	0.167	−0.121 (0.086)	0.159
${\alpha }_{Z2(BMI)}$	0.424 (0.067)	< 0.001	0.304 (0.089)	< 0.001
${\alpha }_{X}$	0.568 (0.093)	< 0.001	0.696 (0.116)	< 0.001
$var({u}_{Wi})$	0.260 (0.055)	< 0.001	0.397 (0.086)	< 0.001
$var({e}_{Wij})$ ^b^	0.151 (0.026)	< 0.001	0.248 (0.047)	< 0.001
$var({e}_{Mij})$	1.551 (0.189)	< 0.001	1.692 (0.212)	< 0.001
${\rho }_{XW}$ ^c^	0.627 (0.073)	< 0.001	0.623 (0.075)	< 0.001
${\rho }_{XM}$	0.589 (0.063)	< 0.001	0.578 (0.066)	< 0.001
${\rho }_{{D}_{X}{D}_{W}}$ ^d^	0.568 (0.103)	< 0.001	0.588 (0.104)	< 0.001
${\rho }_{{D}_{X}{D}_{M}}$	0.354 (0.073)	< 0.001	0.372 (0.073)	< 0.001

^a^ Measurement error models (2) and (3) in the text for biomarker (${M}_{ij}$) and accelerometer (${W}_{ij}$):

$${M}_{ij}={X}_{ij}+{e}_{Mij}$$,

$${W}_{ij}={\alpha }_{0}+{{\varvec{\upalpha}}}_{Z}^{\prime}{\mathbf{Z}}_{i}+{\alpha }_{X}{X}_{ij}+{u}_{Wi}+{e}_{Wij}$$

where ${X}_{ij}$ is true PAEE kcal per day or true PAEE kcal per kg of body mass per day

^b^ $var({e}_{Wij})=$ within-subject error variance when accelerometer is worn for 7 days

^c^ ${\rho }_{XW}=$ partial correlation of ${X}_{ij}$ and ${W}_{ij}$, controlling for study arm (${A}_{i}$) and** Z**_*i*_

${\rho }_{XM}=$ partial correlation of ${X}_{ij}$ and ${M}_{ij}$, controlling for ${A}_{i}$ and** Z**_*i*_

^d^ ${\rho }_{{D}_{X}{D}_{W}}=$ partial correlation of ${D}_{Xij}={X}_{ij}-{X}_{i1}$ and ${D}_{Wij}={W}_{ij}-{W}_{i1}$, controlling for ${A}_{i}$

${\rho }_{{D}_{X}{D}_{M}}=$ partial correlation of ${D}_{Xij}$ and ${D}_{Mij}={M}_{ij}-{M}_{i1}$, controlling for ${A}_{i}$

Table 4 presents parameter estimates for the cLDA intervention model in the BEAT/DLW calibration sub-study for both absolute and body-mass-scaled PAEE. The columns on the left show estimates corrected for measurement error using the combined model (1)-(3), while the columns on the right show uncorrected estimates from a naive model that fits model (1) assuming ${W}_{ij}={X}_{ij}$. Parameters ${\beta }_{TA2}$-${\beta }_{TA4}$ represent the intervention effects at 3, 6 and 12 months after baseline. The estimated effect at the end of 3 months (${\beta }_{TA2}$) is statistically significantly different from zero (p < 0.001) for both absolute and body-mass scaled PAEE and for both the corrected and uncorrected models, but the uncorrected estimated effect is 43% smaller than the corrected effect for absolute PAEE and 30% smaller for body-mass-scaled PAEE. The estimated intervention effects at the end of month 12 suggests that PAEE continues to be greater in the intervention group for up to 9 months after the intervention ends (p < 0.05), although the difference is not statistically significant after adjusting for multiple testing. Overall, the performance of the accelerometer is better for body-mass-scaled PAEE than absolute PAEE.

**Table 4:** Estimated parameters for the cLDA ^a^ intervention models ^b^ in the BEAT/doubly labeled water calibration sub-study, corrected and uncorrected for measurement error (ME) in accelerometry-estimated PAEE; standard errors in parentheses

		Corrected for ME		Uncorrected for ME	
	Parameter	Estimate (s.e.)	*p*-value	Estimate (s.e.)	*p*-value
PAEE	${\beta }_{0}$	−0.231 (0.118)	0.051	0.000 (0.048)	1.000
kcal × d^–1^	${\beta }_{T2}$	0.056 (0.095)	0.560	0.056 (0.056)	0.321
	${\beta }_{T3}$	−0.024 (0.098)	0.810	0.005 (0.057)	0.925
	${\beta }_{T4}$	−0.085 (0.101)	0.398	−0.037 (0.058)	0.529
	${\beta }_{TA2}$	0.490 (0.144)	< 0.001	0.280 (0.075)	< 0.001
	${\beta }_{TA3}$	0.210 (0.132)	0.113	0.124 (0.076)	0.105
	${\beta }_{TA4}$	0.278 (0.138)	0.044	0.180 (0.078)	0.022
	${\beta }_{Z1(Age)}$	−0.014 (0.115)	0.905	−0.101 (0.042)	0.016
	${\beta }_{Z2(BMI)}$	0.258 (0.105)	0.014	0.573 (0.042)	< 0.001
	$var({u}_{Xi})$	0.601 (0.165)	< 0.001	0.444 (0.043)	< 0.001
	$var({\varepsilon }_{Xij})$	0.223 (0.091)	0.012	0.237 (0.012)	< 0.001
PAEE	${\beta }_{0}$	−0.244 (0.124)	0.049	0.007 (0.061)	0.904
kcal × kg^–1^ × d^–1^	${\beta }_{T2}$	0.070 (0.102)	0.495	0.081 (0.073)	0.272
	${\beta }_{T3}$	−0.014 (0.105)	0.891	0.016 (0.075)	0.831
	${\beta }_{T4}$	−0.085 (0.108)	0.429	−0.042 (0.076)	0.579
	${\beta }_{TA2}$	0.535 (0.154)	< 0.001	0.378 (0.098)	< 0.001
	${\beta }_{TA3}$	0.257 (0.142)	0.070	0.190 (0.099)	0.056
	${\beta }_{TA4}$	0.313 (0.148)	0.034	0.243 (0.102)	0.017
	${\beta }_{Z1(Age)}$	0.006 (0.119)	0.957	−0.117 (0.052)	0.024
	${\beta }_{Z2(BMI)}$	−0.363 (0.110)	< 0.001	0.053 (0.052)	0.301
	$var({u}_{Xi})$	0.576 (0.171)	< 0.001	0.657 (0.065)	< 0.001
	$var({\varepsilon }_{Xij})$	0.272 (0.106)	0.017	0.403 (0.021)	< 0.001

^a^ cLDA = constrained Longitudinal Data Analysis (assumes *β*_*A*_ = 0)

^b^ Intervention model (1) in the text: ${X}_{ij}={\beta }_{0}+{\beta }_{A}{A}_{i}+{{\varvec{\upbeta}}}_{T}^{\prime}{\mathbf{T}}_{j}+{{\varvec{\upbeta}}}_{TA}^{\prime}{\mathbf{T}}_{j}{A}_{i}+{{\varvec{\upbeta}}}_{Z}^{\prime}{\mathbf{Z}}_{i}+{u}_{Xi}+{\varepsilon }_{Xij}$,

where ${X}_{ij}$ is true PAEE kcal per day or true PAEE kcal per kg of body mass per day

**Fig. 2:**
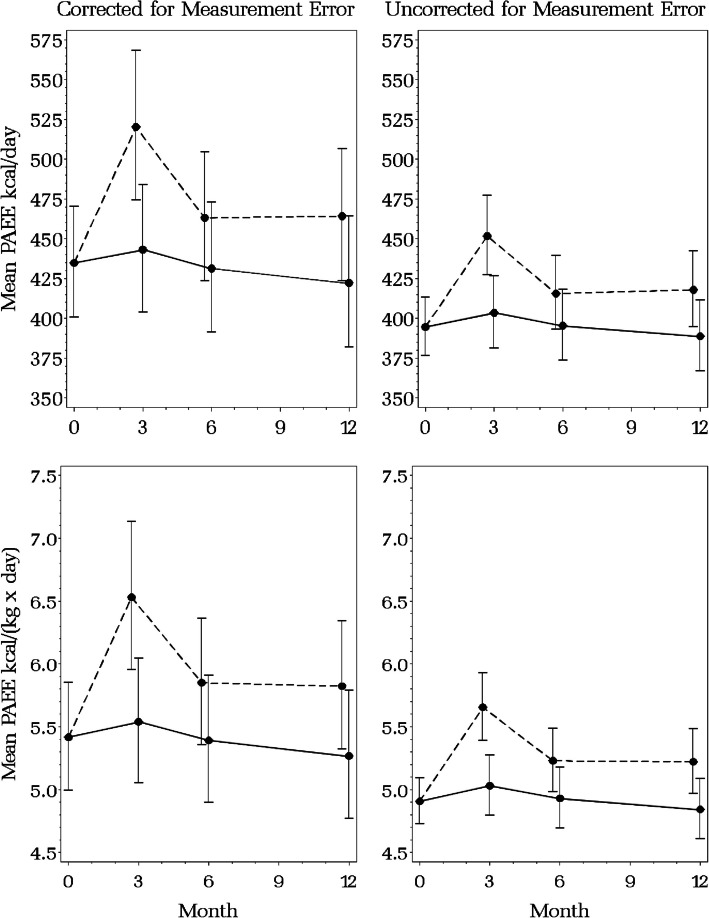
Estimated mean physical activity energy expenditure (PAEE) at months 0 (baseline), 3 (end of intervention), 6 and 12, corrected (left panel) and uncorrected (right panel) for measurement error in accelerometry-estimated PAEEPAEE kcal × d^–1^ (upper panels) and PAEE kcal × kg^–1^ × d^–1^ (lower panels). Solid line = usual care arm with 95% confidence interval, Dashed line = BEAT Cancer intervention arm with 95% confidence interval

**Fig. 3:**
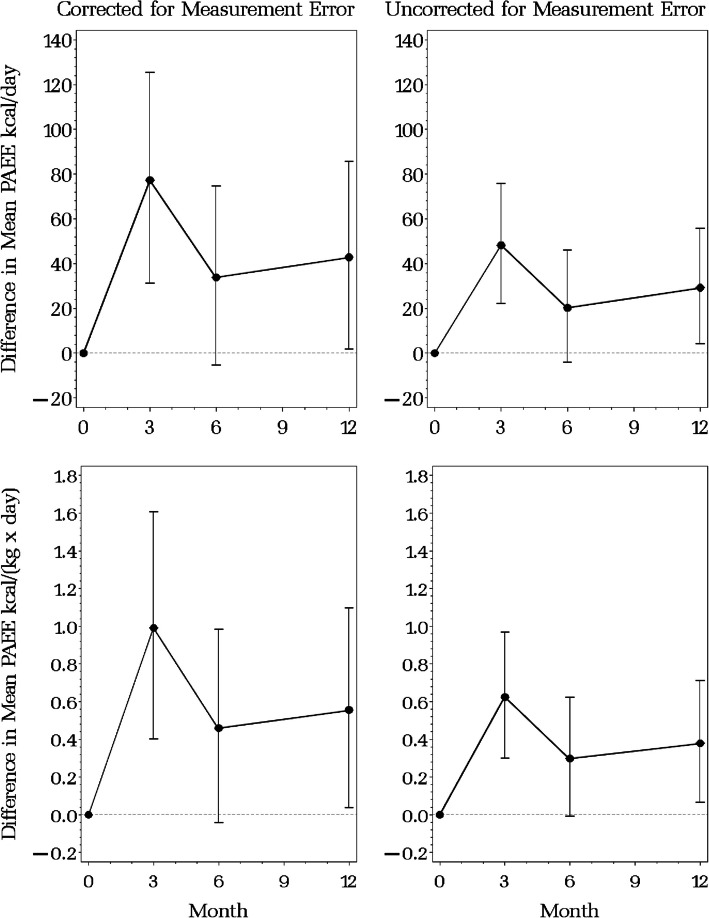
Difference in estimated mean physical activity energy expenditure (PAEE) between arms (BEAT – usual care) at months 0 (baseline), 3 (end of intervention), 6 and 12, with 95% confidence interval, corrected (left panel) and uncorrected (right panel) for measurement error in accelerometry-estimated PAEE kcal × d^–1^ (upper panels) and PAEE kcal × kg^–1^ × d.^–1^ (lower panels)

The estimated intervention effects ${\beta }_{TA2}$-${\beta }_{TA4}$ in Table 4 are for the transformed and standardized response variables $X=\left(\sqrt{PAEE}-Mean\sqrt{PAEE}\right) / Std Dev \sqrt{PAEE}$ and $W=\left\{\text{log}PAEE-Mean(\text{log}PAEE)\right\} / Std Dev(\text{log}PAEE)$. The model parameters can also be used to estimate intervention effects on the original scale. Figure 2 presents the estimated mean PAEE (absolute and scaled for body mass) and 95% confidence interval on the original scale by study arm at months 0, 3, 6 and 12, corrected and uncorrected for measurement error in the accelerometer. The overlapping confidence intervals for the intervention and usual care arms are due to the high correlation between the point estimates and do not indicate a lack of statistically significant differences. Figure 3 presents the estimated intervention effect (difference between arms) and 95% confidence intervals (CI) on the original scale for the estimated difference between the two arms. The estimated intervention effect is statistically significant at month 3 for both the corrected and uncorrected models (the 95% CI does not include zero), although the uncorrected effect is about 40% smaller than the corrected effect for both absolute and body-mass-scaled PAEE. For absolute PAEE, the corrected and uncorrected estimated intervention effects are 77 (95% CI = 22–126) and 48 (95% CI = 28–75), respectively, while for body-mass-scaled PAEE, they are 0.99 (95% CI = 0.40–1.61) and 0.62 (95% CI = 0.30–0.97).

For comparison, we also fitted a model based on the biomarker only, which is available only in the calibration sub-study (*n* = 82). Supplemental Figure A1 (Additional File 3) in the online supplementary material shows the estimated mean PAEE (kcal × d^–1^ and kcal × kg^–1^ × d^–1^) by treatment arm, and the estimated difference in mean PAEE between arms. The means by treatment arm show no improvement over time in either arm (PAEE actually declines), and the difference between arms is not statistically significantly different from zero at any time point. The lack of significant improvement over time in the treatment arm is due not only to the small sample size but also to the large within-subject variation in the biomarker, $var({e}_{Mij})$*,* shown in Table 3, and the resulting low correlation between true and measured difference from baseline, ${\rho }_{{D}_{X}{D}_{M}}$. This is a case where the biomarker, though unbiased, is less sensitive than the accelerometer (${\rho }_{{D}_{X}{D}_{M}}<{\rho }_{{D}_{X}{D}_{W}})$, offering an opportunity to improve estimation by combining the instruments in a structural model.

The cLDA model assumes that mean PAEE at baseline (prior to intervention) does not differ by study arm, an assumption that should be true in a properly randomized trial. As a sensitivity analysis, we also fitted the LDA model that allows mean PAEE to differ by study arm at baseline. Table A1 in the online supplemental material presents parameter estimates for the LDA model. The estimated difference at baseline for absolute PAEE, ${\widehat{\beta }}_{A}=0.047$ (standard error $=0.160$), and for body-mass-scaled PAEE ${\widehat{\beta }}_{A}=0.004$ (standard error = 0.165), is small and not statistically significantly different from zero (*p* > 0.75). Comparing Tables 4 and A1, we see that estimated intervention effects for the LDA and cLDA models are similar, but the standard errors of the cLDA estimates are smaller than those of the LDA estimates.

As discussed in the previous section, assuming incorrectly that measurement error is nondifferential can lead to biased estimation of intervention effects. We looked for evidence of differential error due to BMI or cardiorespiratory fitness (${F}_{ij}$) by fitting the differential error model described in the previous section. Table A2 in the online supplemental material presents the results for absolute PAEE. Under the model, error is differential if ${{\varvec{\upgamma}}}_{TA}\ne 0$ and ${\alpha }_{F}\ne 0$. For cardiorespiratory fitness, ${{\varvec{\upgamma}}}_{TA}$ was statistically significantly different from zero at months 6 (*p* = 0.007) and 12 (*p* = 0.034) but *α*_*F*_ was not (*p* = 0.135). For BMI, neither ${{\varvec{\upgamma}}}_{TA}\ne 0$ (*p* > 0.22) nor ${\alpha }_{F}\ne 0$ (*p* = 0.086) were statistically significantly different from zero. In addition, including BMI or fitness in the model had little effect on the estimated intervention effects (${{\varvec{\upbeta}}}_{TA}$). A similar analysis for body mass scaled PAEE gave similar results and led to similar conclusions (results not shown). We also considered a more general model of differential error that fits model (3) to absolute PAEE (but not body mass scaled PAEE) separately within each study arm. A likelihood ratio test did not find this stratified model to fit better than model (3) (*p* = 0.86). Overall, we found little evidence that measurement error was differential in this trial.

## Discussion

To our knowledge, this is the first randomized physical activity intervention trial that has measured objective and unbiased TEE and REE longitudinally in a sub-study of participants. This allowed us to evaluate measurement error in accelerometry-derived estimates of short-term PAEE and changes over time in this setting and correct for bias due to measurement error. Our analysis found that measurement error in accelerometry-estimated PAEE included systematic bias related to both true PAEE and baseline BMI, and that ignoring such error can lead to bias in estimated intervention effects. After correcting for bias due to measurement error in our study, the estimated intervention effect at month 3 was an increase in absolute PAEE of 77 kcal/day (95% CI = 22–126), compared to 48 kcal/day (95% CI = 28–75) when measurement error was ignored. These results indicate a 20% (21%) increase in PAEE kcal x d^−1^ (kcal x kg^−1^ × d^−1^) at month 3 relative to baseline for the corrected model vs. 14% (15%) for the uncorrected model, demonstrating that the greater intervention effects noted with correction for measurement error was due to a greater percent increase and not simply due to a higher baseline PAEE. Scaling PAEE for body mass improved the accelerometer performance (see Tables 3 and 4), supporting the use of this metric in intervention trials and further supporting the consideration of BMI when measuring and analyzing absolute PAEE in a sample with high variability in BMI. Replication of these results in other populations could have significant practical and public health impacts with regard to research testing PAEE response to interventions and associations with health risk, especially for primary outcomes such as body weight and disease biomarkers.

The study design also provided the opportunity to determine whether the error in accelerometry-estimated PAEE was differential, which has not previously been examined in an intervention trial. We found no evidence of differential error in our trial, but this does not preclude the possibility of differential error in other trials, particularly if they involve other physical activity interventions. The fact that baseline BMI was one of the factors related to systematic bias in accelerometry-estimated PAEE suggests that differential error might be a problem in trials with interventions designed to produce weight change, especially if not scaled for body mass. This is consistent with the fact that excess body weight reduces accelerometer accuracy through factors that change with weight loss, including altered gait mechanics (and resultant energy expenditure) and accelerometer placement challenges relative to the center of body mass [14, 37, 38].

Given the importance of body mass for PAEE, we also ran alternative models that scaled PAEE for body mass (PAEE/kilograms of body weight). Our overall conclusion that the BEAT intervention increased physical activity in the trial did not depend on whether or not we corrected for measurement error or whether we used absolute or body-mass-scaled PAEE as our physical activity metric. Looking at the size of the estimated intervention effects, we did see that the uncorrected estimates were attenuated relative to the measurement-error-corrected estimates. In this respect, using body-mass-scaled PAEE improved the performance of the accelerometer by reducing the bias.

We recommend that intervention trials using an accelerometer to measure physical activity consider including a calibration sub-study in which DLW and REE are also measured. If such a sub-study is not feasible due to cost or local capacity, we recommend performing a sensitivity analysis with and without correcting for measurement error, using the parameters identified here. Acknowledging the major contribution of body weight to absolute PAEE and consistent with our measurement results reported here, scaling by body mass when testing intervention effects on PAEE may be especially important in trials in which the change in BMI differs by study condition or includes samples that vary greatly in body size. This would be particularly important for studies with primary outcomes such as weight loss, fatigue, and/or biological markers because of the greater relevance of PAEE over exercise behavior alone.

Although no prior study has evaluated the structure of measurement error in accelerometer-estimated physical activity when used as the outcome of a randomized intervention trial, two prior reports that used DLW to assess measurement error in cross-sectional, observational studies warrant comparison. In contrast to that reported by Matthews et al. [10], we found that BMI is associated with systematic accelerometer measurement error, a finding with particularly relevant implications for interventions combining physical activity with weight loss. This inconsistency may be due, in part, to differences in study design (e.g., longitudinal vs. cross-sectional) and sample characteristics (e.g., our sample included women only who were, on average, younger with higher BMI). Although Agogo et al. [9] reported underestimation of TEE measurement using the accelerometer (a finding consistent with our larger intervention effect after measurement error correction), measurement error related to PAEE was not reported nor was testing of the error longitudinally done. It should also be noted that both studies relied on prediction equations to estimate REE rather than measuring it by indirect calorimetry.

The validity of our analysis and conclusions relies on the assumption that models (1)-(3) provide a good approximation of the joint distribution of true and estimated PAEE. We have argued that the assumption of an (approximately) unbiased biomarker is well-established and considered alternative models to investigate the possibility that mean PAEE at baseline differs between the two arms or that measurement error in the accelerometer is differential but have not ruled out other possible sources of model misspecification.

We acknowledge several limitations. First, the differences in fitness levels suggested as potentially present based our preliminary work [13] may not have been detected due to our small sample size or the use of prediction equations when measuring our fitness outcome. Also, the BEAT Cancer study was not designed to elicit weight loss thus additional research is needed to assess potential measurement error (and the error structure) that occurs among individuals participating in a randomized weight loss trial. Another limitation is the lack of an unbiased reference biomarker of MVPA, the primary outcome of the original BEAT Cancer trial. Thus, it is not possible to directly compare our reporting here of continued statistically significant intervention benefit on PAEE at 12 months (9 months after intervention completion) with MVPA. However, our PAEE findings are consistent with the statistically significant intervention fitness benefits reported at 12 months [15]. Also, we used a standard protocol to process the accelerometer data and estimate PAEE, but we did not consider any alternative methods that might have improved the accelerometer-based estimates. Although our results may be generalizable to women with a history of cancer type other than breast who are in remission well after treatment, our results are less generalizable to individuals with cancer-related factors that may influence REE and TEE, including treatment type (e.g., chemotherapy) and progressive cancer types (e.g., pancreatic, liver, and lung). However, the striking difference with and without error adjustment (nearly 50% reduction in intervention effect) indicates the high value of repeating this study in other populations.

This study had several significant strengths including the incorporation of a calibration sub-study within an intervention trial, repeated measurements over the course of a year, and the focus on measurement error in the trial outcome rather than a covariate. The study design exemplifies the rigorous approach to measurement error analysis sometimes seen in other fields such a nutrition and extends its application from observational studies to intervention trials. This report also moves beyond self-report to focus on accelerometer-related measurement error, identifying the error as nondifferential (compared to the differential error seen with self-report) and describing the measurement error impact on intervention effect on PAEE.

## Conclusions

This study provides strong evidence of systematic and person-specific measurement error in accelerometer-estimated PAEE. It also demonstrates how ignoring such errors can bias the results of a randomized trial, in our case leading to a reduction in the estimated intervention effect. If feasible, calibration sub-studies with unbiased reference measures should be incorporated into physical activity intervention trials that use accelerometers to measure the primary outcome; otherwise, sensitivity analyses should be performed to assess the potential impact of measurement error. Although we found no evidence of differential measurement error in our trial, we did find that measurement error in accelerometry-estimated PAEE differs by baseline BMI and by whether PAEE is scaled for body mass. This suggests that trials of physical activity interventions that could substantially impact weight should consider scaling PAEE for body mass. Further study with newer objective motion sensors along with innovative arrays of accelerometers worn simultaneously on different body segments is needed to assess the generalizability of our findings to other instruments and populations and especially to weight loss trials. Doing so along with including more precise measures of diet-induced thermogenesis from diet data along with portable or room calorimetry would also enhance understanding of how to optimally apply absolute PAEE by DLW measurements to assessing measurement error in populations with widely varying body mass.

## Supplementary Information

Additional file 1. CONSORT checklist_v2.

Additional file 2. supplemental analytic detail.

Additional file 3. Supplemental.Figure.A1.

## Data Availability

Data and materials available from the corresponding author on reasonable request.

## Abbreviations

PAEE: Physical activity energy expenditure
BEAT Cancer: Better Exercise Adherence after Treatment for Cancer intervention
TEE: Total energy expenditure
REE: Resting energy expenditure
COMPARE: Comparing doubly labeled water to accelerometer to assess physical activity measurement error during and after a physical activity behavior change intervention
DLW: Doubly labeled water
CONSORT: Consolidated standards of reporting trials
BMI: Body mass index
LDA: Unconstrained longitudinal data analysis
ANCOVA: Analysis of covariance
cLDA: Constrained longitudinal data analysis
IQR: Interquartile range
DCIS: Ductal carcinoma in situ
s.e.: Standard error
ME: Measurement error
