# Supplementary material for: Accelerometer measurement error in a randomized physical activity intervention trial in breast cancer survivors was nondifferential but attenuated the intervention effect
Source: Int J Behav Nutr Phys Act. 2025 May 26;22:59. doi: 10.1186/s12966-025-01760-5 (PMC12105316; doi:10.1186/s12966-025-01760-5)
Supplement: Supplementary file 2 — Additional file 2. supplemental analytic detail. [file 12966_2025_1760_MOESM2_ESM.docx]

**Accelerometer measurement error in a randomized physical activity intervention trial in breast cancer survivors was nondifferential but attenuated the intervention effect.**

**Additional File 2 – Supplemental Analytic Detail**

**A1: Change score and ANCOVA methods**

The LDA and cLDA models described in the main text are closely related to the change score and ANCOVA methods commonly used in intervention studies. Under the intervention model (1) in the main text, the change from baseline $D_{Xij} =X_{ij}-X_{i1}$ has the following form,

$D_{Xij}=\boldsymbol{\beta}_{T}^{'}\mathbf{T}_{j}+\boldsymbol{\beta}_{TA}^{'}\mathbf{T}_{j}A_{i}+\delta_{Xij}$, *j* = 2, 3, …, *J*,

where $\delta_{Xij}=\varepsilon_{Xij}-\varepsilon_{Xi1}$ has variance $var(\delta_{Xij})=2\sigma_{\varepsilon_{X}}^{2}$. The change score method estimates $\beta_{TAj}$ as $\hat{\beta}_{TAj}=\bar{D}_{Xj}^{(1)}-\bar{D}_{Xj}^{(0)}$, where $\bar{D}_{Xj}^{(k)}$ is the mean change from baseline at time *j* in arm *k*. The standard error of $\hat{\beta}_{TAj}$ is $se\left( \hat{\beta}_{TAj} \right)=\sqrt{2\sigma_{\varepsilon_{X}}^{2}(1/N_{1}+1/N_{2})}$, where *N_k_* is the number of subjects in arm *k*. When there are no missing data, the estimated intervention effects for the change score and LDA models are identical [27].

The longitudinal ANCOVA model [28] is a multivariate linear regression model that includes baseline response $X_{i1}$ as a covariate,

$D_{Xij}=\boldsymbol{\beta}_{T}^{'}\mathbf{T}_{j}+\boldsymbol{\beta}_{TA}^{'}\mathbf{T}_{j}A_{i}+\gamma_{Xj}X_{i1}+\nu_{Xij}$, *j* = 2, 3, …, *J*,

where $\nu_{Xij}$ has variance $var(\nu_{Xij})=var(\delta_{Xij})\left\{ 1-{corr}^{2}(\delta_{Xij},X_{i1}) \right\}=var(\delta_{Xij})(1+\rho_{X_{j}X_{1}})/2$, and $\rho_{X_{j}X_{1}}=corr\left( X_{ij},X_{i1} | A_{i},\mathbf{Z}_{i} \right)$ is the partial correlation of $X_{ij}$ and $X_{i1}$ controlling for $A_{i}$ and $\mathbf{Z}_{i}$ [27]. The standard error of the ANCOVA estimator $\hat{\beta}_{TAj}^{ANC}$ is $se\left( \hat{\beta}_{TAj}^{ANC} \right)=se\left( \hat{\beta}_{TAj} \right)\sqrt{(1+\rho_{X_{j}X_{1}})/2}$. It follows that the ANCOVA estimator is more efficient than the change score estimator.

When there are no missing data and the longitudinal ANCOVA model is fit assuming $\boldsymbol{\nu}_{Xi}=(\nu_{Xi2},\ldots,\nu_{XiJ})'$ has a compound symmetric covariance matrix, the longitudinal ANCOVA model and LDA model give identical estimates of the intervention effects [28]. Combining results, we can conclude that the cLDA estimator is more efficient than the LDA estimator.

**A2: Naïve change score method**

Under measurement error model (3) in the main text, the observed change from baseline $D_{Wij} =W_{ij}-W_{i1}$ has the following form,

$D_{Wij}=\alpha_{X}D_{Xij}+\delta_{Wij}$,

where $\delta_{Wij}=e_{Wij}-e_{Wi1}$ has variance $var(\delta_{Wij})=2(\alpha_{X}^{2}\sigma_{\varepsilon_{X}}^{2}+\sigma_{e_{W}}^{2})$.

If $W_{ij}$ is observed, a naïve change score method would estimate $\beta_{TAj}$ as $\hat{\beta}_{TAj}^{*}=\bar{D}_{Wj}^{(1)}-\bar{D}_{Wj}^{(0)}$, where $\bar{D}_{Wj}^{(k)}$ is the mean change in $W_{ij}$ from baseline in arm *k*. The mean and standard error of $\hat{\beta}_{TAj}^{*}$ would be $E(\hat{\beta}_{TAj}^{*})=\alpha_{X}\beta_{TAj}$ and $se\left( \hat{\beta}_{TAj}^{*} \right)=\sqrt{2\left( \alpha_{X}^{2}\sigma_{\varepsilon_{X}}^{2}+\sigma_{e_{W}}^{2} \right)/(1/N_{1}+1/N_{2})}$. We note that when $W_{ij}$ has classical measurement error, $\alpha_{X}=1$ and the naïve estimator is consistent (asymptotically unbiased).

**A3: Loss of power to detect an intervention effect**

We illustrate the loss of power due to measurement error when intervention effects are estimated using the naive change score method described above. The standard error for this method has a particularly simple form, making it easy to illustrate results.

Consider a hypothetical intervention trial in which true outcome $X_{ij}$ is observed on *N* participants (*N_k_* = *N* / 2 in each arm). The null hypothesis that there is no intervention effect at time *j* ($\beta_{TAj}=0$) could be tested using the z-statistic $z_{X}={\hat{\beta}_{TAj}}/{\hat{\mathrm{se}}\left( \hat{\beta}_{TAj};N \right)}$, where $\hat{\mathrm{se}}\left( \hat{\beta}_{TAj};N \right)$ is the estimated standard error of $\hat{\beta}_{TAj}$. Since $z_{X}$ is asymptotically normal with unit variance, the power to reject the null hypothesis depends on the absolute value of the mean $|E\left( z_{X};N \right)|$ as *N* → ∞, where

$E\left( z_{X};N \right)\approx{E(\hat{\beta}_{TAj})}/{se\left( \hat{\beta}_{TAj};N \right)}={\beta_{TAj}}/{\sqrt{8\sigma_{\varepsilon_{X}}^{2}/N}}$.

If $W_{ij}$ is observed instead of $X_{ij}$, the z-statistic for the naïve change score method would have mean

$$E\left( z_{W};N \right)\approx{E(\hat{\beta}_{TAj}^{*})}/{se\left( \hat{\beta}_{TAj}^{*};N \right)}={\alpha_{X}\beta_{TAj}}/{\sqrt{8\left( \alpha_{X}^{2}\sigma_{\varepsilon_{X}}^{2}+\sigma_{e_{W}}^{2} \right)/N}}=E\left( z_{X};N^{*} \right)$$

where $\rho_{D_{X}D_{W}}^{2}=\alpha_{X}^{2}\sigma_{\varepsilon_{X}}^{2}/(\alpha_{X}^{2}\sigma_{\varepsilon_{X}}^{2}+\sigma_{e_{W}}^{2})$ is the squared partial correlation of $D_{Xij}$ and $D_{Wij}$ controlling for $A_{i}$, and $N^{*}=\rho_{D_{X}D_{W}}^{2}N$ is the “effective sample size” of the naive test. Since both z-statistics have mean zero under the null hypothesis, both provide valid tests of the null hypothesis. Since $\rho_{D_{X}D_{W}}^{2}\leq1$ implies that $N^{*}\leq N$, however, with equality if and only if $\sigma_{e_{W}}^{2}=0$, the test based on $z_{W}$ is less powerful than the test based on $z_{X}$,. To have the same power as a test based on $z_{X}$ with *N* participants, the test based on $z_{W}$ would need a sample size of $N^{W}=N/\rho_{D_{X}D_{W}}^{2}$.

**Table A1: Estimated parameters for the LDA^a^ intervention model^b^ in the BEAT study.**

|  | PAEE kcal × d^–1^ | | PAEE kcal × kg^–1^ × d^–1^ | |
| --- | --- | --- | --- | --- |
| Parameter | **Estimate (s.e.^c^)** | **p-value** | **Estimate (s.e.)** | **p-value** |
|  |  |  |  |  |
| $\boldsymbol{\beta}_{\boldsymbol{0}}$ | -0.256 (0.145) | 0.079 | -0.246 (0.151) | 0.104 |
| $\boldsymbol{\beta}_{\boldsymbol{T}\boldsymbol{2}}$ | 0.064 (0.100) | 0.519 | 0.071 (0.108) | 0.512 |
| $\boldsymbol{\beta}_{\boldsymbol{T}\boldsymbol{3}}$ | -0.015 (0.102) | 0.886 | -0.013 (0.110) | 0.903 |
| $\boldsymbol{\beta}_{\boldsymbol{T}\boldsymbol{4}}$ | -0.076 (0.105) | 0.468 | -0.084 (0.113) | 0.456 |
|  |  |  |  |  |
| $\boldsymbol{\beta}_{\boldsymbol{A}}$ | 0.047 (0.160) | 0.768 | 0.004 (0.165) | 0.979 |
| $\boldsymbol{\beta}_{\boldsymbol{TA}\boldsymbol{2}}$ | 0.472 (0.154) | 0.002 | 0.533 (0.169) | 0.002 |
| $\boldsymbol{\beta}_{\boldsymbol{TA}\boldsymbol{3}}$ | 0.192 (0.144) | 0.181 | 0.255 (0.157) | 0.104 |
| $\boldsymbol{\beta}_{\boldsymbol{TA}\boldsymbol{4}}$ | 0.261 (0.149) | 0.080 | 0.311 (0.162) | 0.055 |
|  |  |  |  |  |
| $\boldsymbol{\beta}_{\boldsymbol{Z1(Age)}}$ | -0.013 (0.115) | 0.912 | 0.007 (0.119) | 0.956 |
| $\boldsymbol{\beta}_{\boldsymbol{Z}\boldsymbol{2(BMI)}}$ | 0.258 (0.105) | 0.014 | -0.363 (0.110) | < 0.001 |
|  |  |  |  |  |
| $\boldsymbol{var(}\boldsymbol{u}_{\boldsymbol{Xi}}\boldsymbol{)}$ | 0.600 (0.164) | < 0.001 | 0.576 (0.171) | < 0.001 |
| $\boldsymbol{var(}\boldsymbol{\varepsilon}_{\boldsymbol{Xij}}\boldsymbol{)}$ | 0.222 (0.091) | 0.012 | 0.272 (0.106) | 0.011 |

^a^ LDA = Longitudinal Data Analysis.

^b^ Intervention model (1) in the text: $X_{ij}=\beta_{0}+\beta_{A}A_{i}+\boldsymbol{\beta}_{T}^{'}\mathbf{T}_{j}+\boldsymbol{\beta}_{TA}^{'}\mathbf{T}_{j}A_{i}+\boldsymbol{\beta}_{Z}^{'}\mathbf{Z}_{i}+u_{Xi}+\varepsilon_{Xij}$,

where $X_{ij}$ is true PAEE kcal per day or true PAEE kcal per kg of body mass per day

^c^ s.e. = standard error.

**Table A2: Estimated parameters for the cLDA^a^ model for PAEE kcal × d^–1^ in the BEAT study when baseline and change from baseline BMI or cardiovascular fitness are included as covariates in the measurement error model.**

|  |  | BMI | | Fitness | |
| --- | --- | --- | --- | --- | --- |
| Variable | **Parameter** | **Estimate (s.e.^b^)** | **p-value** | **Estimate (s.e.)** | **p-value** |
|  |  |  |  |  |  |
| $\boldsymbol{X}_{\boldsymbol{ij}}$ | $\boldsymbol{\beta}_{\boldsymbol{0}}$ | -0.266 (0.117) | 0.023 | -0.232 (0.115) | 0.043 |
|  | $\boldsymbol{\beta}_{\boldsymbol{T}\boldsymbol{2}}$ | 0.050 (0.095) | 0.596 | 0.031 (0.099) | 0.754 |
|  | $\boldsymbol{\beta}_{\boldsymbol{T}\boldsymbol{3}}$ | -0.052 (0.098) | 0.597 | -0.057 (0.101) | 0.571 |
|  | $\boldsymbol{\beta}_{\boldsymbol{T}\boldsymbol{4}}$ | -0.095 (0.101) | 0.346 | -0.104 (0.104) | 0.317 |
|  |  |  |  |  |  |
|  | $\boldsymbol{\beta}_{\boldsymbol{TA}\boldsymbol{2}}$ | 0.494 (0.143) | < 0.001 | 0.496 (0.145) | < 0.001 |
|  | $\boldsymbol{\beta}_{\boldsymbol{TA}\boldsymbol{3}}$ | 0.229 (0.132) | 0.083 | 0.211 (0.134) | 0.116 |
|  | $\boldsymbol{\beta}_{\boldsymbol{TA}\boldsymbol{4}}$ | 0.284 (0.138) | 0.040 | 0.270 (0.140) | 0.054 |
|  |  |  |  |  |  |
|  | $\boldsymbol{\beta}_{\boldsymbol{Z}\boldsymbol{1(Age)}}$ | -0.029 (0.115) | 0.800 | -0.055 (0.115) | 0.629 |
|  | $\boldsymbol{\beta}_{\boldsymbol{Z}\boldsymbol{2(BMI)}}$ | * | * | 0.313 (0.105) | 0.003 |
|  |  |  |  |  |  |
| $\boldsymbol{F}_{\boldsymbol{ij}}$^c^ | $\boldsymbol{\gamma}_{\boldsymbol{0}}$ | -0.004 (0.057) | 0.945 | -0.011 (0.051) | 0.824 |
|  | $\boldsymbol{\gamma}_{\boldsymbol{T}\boldsymbol{2}}$ | 0.008 (0.016) | 0.629 | 0.320 (0.060) | < 0.001 |
|  | $\boldsymbol{\gamma}_{\boldsymbol{T}\boldsymbol{3}}$ | 0.0003 (0.016) | 0.984 | 0.204 (0.066) | 0.002 |
|  | $\boldsymbol{\gamma}_{\boldsymbol{T}\boldsymbol{4}}$ | 0.0002 (0.017) | 0.992 | 0.251 (0.069) | < 0.001 |
|  |  |  |  |  |  |
|  | $\boldsymbol{\gamma}_{\boldsymbol{TA}\boldsymbol{2}}$ | -0.027 (0.022) | 0.223 | 0.120 (0.080) | 0.134 |
|  | $\boldsymbol{\gamma}_{\boldsymbol{TA}\boldsymbol{3}}$ | -0.027 (0.023) | 0.230 | 0.238 (0.089) | 0.007 |
|  | $\boldsymbol{\gamma}_{\boldsymbol{TA}\boldsymbol{4}}$ | -0.015 (0.023) | 0.530 | 0.199 (0.094) | 0.034 |
|  |  |  |  |  |  |
|  | $\boldsymbol{\gamma}_{\boldsymbol{Z}\boldsymbol{1(Age)}}$ | 0.006 (0.057) | 0.917 | -0.199 (0.045) | < 0.001 |
|  | $\boldsymbol{\gamma}_{\boldsymbol{Z2(BMI)}}$ | * | * | -0.406 (0.045) | < 0.001 |
|  |  |  |  |  |  |
| $\boldsymbol{W}_{\boldsymbol{ij}}$ | $\boldsymbol{\alpha}_{\boldsymbol{0}}$ | 0.158 (0.062) | 0.011 | 0.134 (0.061) | 0.027 |
|  | $\boldsymbol{\alpha}_{\boldsymbol{Z}\boldsymbol{1(Age)}}$ | -0.092 (0.065) | 0.154 | -0.080 (0.063) | 0.206 |
|  | $\boldsymbol{\alpha}_{\boldsymbol{Z}\boldsymbol{2(BMI)}}$ | * | * | 0.458 (0.074) | < 0.001 |
|  | $\boldsymbol{\alpha}_{\boldsymbol{X}}$ | 0.550 (0.088) | < 0.001 | 0.536 (0.089) | < 0.001 |
|  | $\boldsymbol{\alpha}_{\boldsymbol{F}\boldsymbol{1}}$ | 0.478 (0.069) | < 0.001 | -0.016 (0.060) | 0.789 |
|  | $\boldsymbol{\alpha}_{\boldsymbol{F}}$ | -0.054 (0.032) | 0.086 | 0.048 (0.032) | 0.135 |

^a^ cLDA = constrained Longitudinal Data Analysis.

^b^ s.e. = standard error.

^c^ $F_{ij}=$ log BMI or Fitness.

* Since $F_{ij}=$ log BMI, $\beta_{Z2}$, $\gamma_{Z2}$ and $\alpha_{Z2}$ do not appear in this model.
