# Supplementary material for: Accelerometer measurement error in a randomized physical activity intervention trial in breast cancer survivors was nondifferential but attenuated the intervention effect
Source: Int J Behav Nutr Phys Act. 2025 May 26;22:59. doi: 10.1186/s12966-025-01760-5 (PMC12105316; doi:10.1186/s12966-025-01760-5)

**Supplemental Figure A1**: Estimates based on the biomarker only (n = 82). **Left panels**: Estimated mean physical activity energy expenditure (PAEE) by treatment arm (solid line = usual care, dashed line = BEAT Cancer intervention) for kcal×d^–1^ (upper panel) and kcal×kg^–1^×d^–1^ (lower panel), with 95% confidence intervals. **Right panels**: Estimated difference in mean PAEE between arms (BEAT – usual care) for kcal×d^–1^ (upper panel) and kcal×kg^–1^×d^–1^ (lower panel), with 95% confidence intervals.


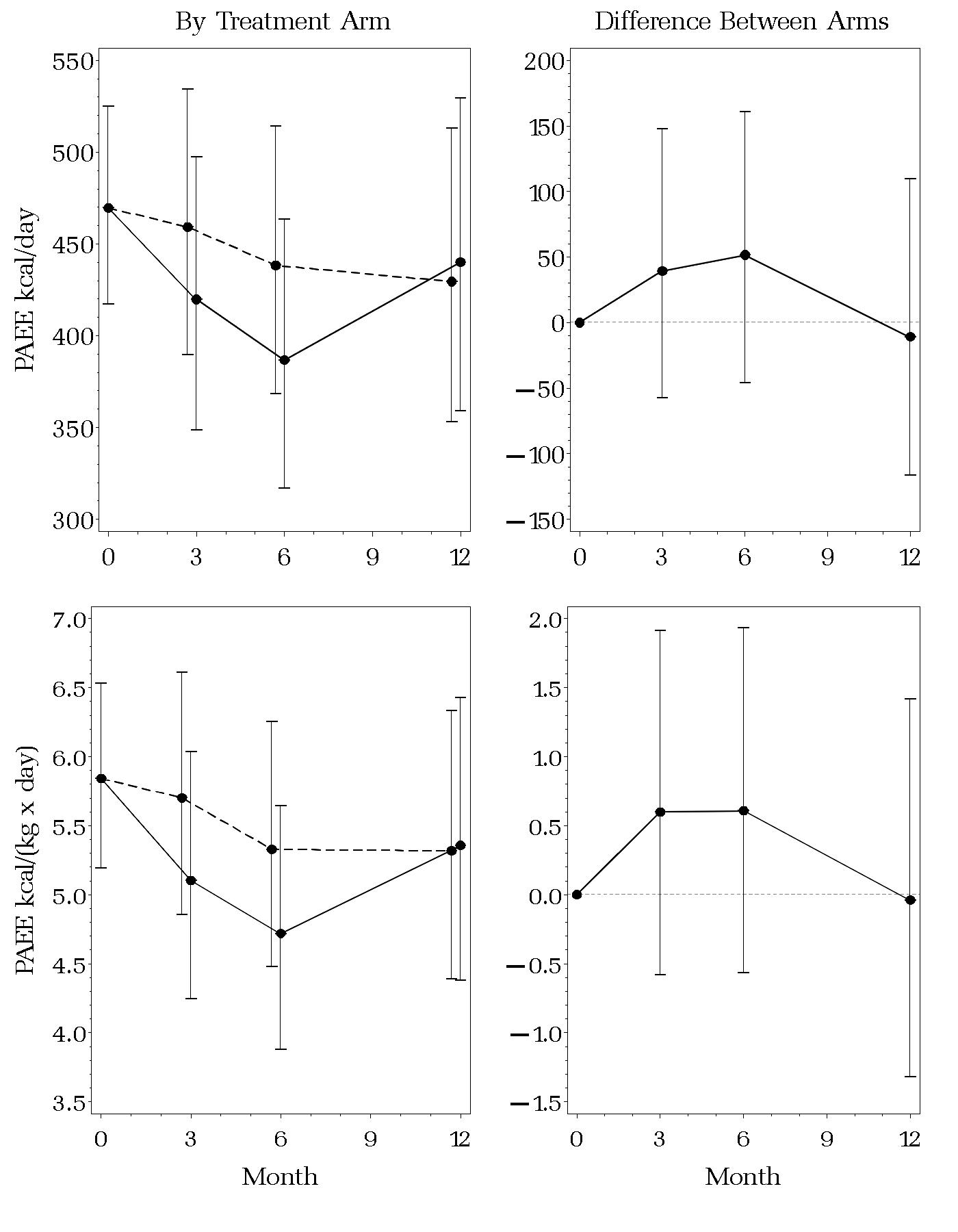

Supplement: Supplementary file 3 — Additional file 3. Supplemental.Figure.A1. [file 12966_2025_1760_MOESM3_ESM.docx]
